# Supplementary material for: Contributions of adaptation and purifying selection to SARS-CoV-2 evolution
Source: Virus Evol. 2022 Dec 10;8(2):veac113. doi: 10.1093/ve/veac113 (PMC10431346; doi:10.1093/ve/veac113)
Supplement: veac113_Supp [file veac113_supp.zip › supplementary_figures.pdf]

Contributions of adaptation and purifying selection to SARS-CoV-2 evolution Supplementary Figures

Richard A. Neher

Biozentrum, University of Basel, Basel, Switzerland and Swiss Institute of Bioinformatics, Switzerland

(Dated: November 20, 2022)

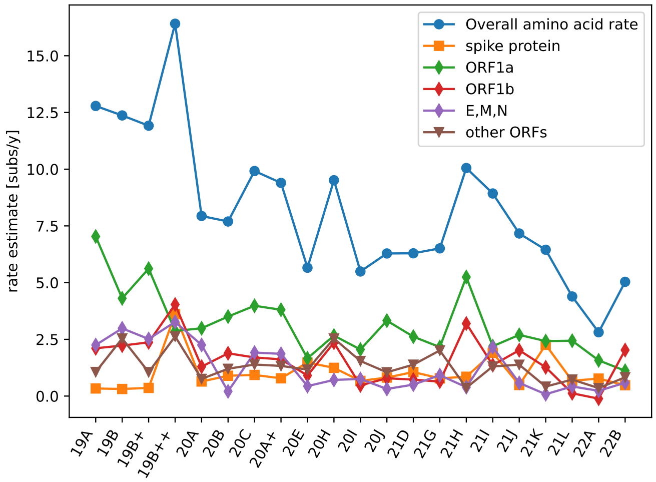

Figure S 1 **Within-clade non-synonymous evolution in different parts of the genome.** The decreasing trend in the within-clade non-synonymous rate of evolution is most evident in the largest *ORF1ab*. Accessory proteins and N,E,M show a weak trend. Clades are ordered alphabetically, which corresponds to their order of designation, which in turn is very similar to their order of emergence.

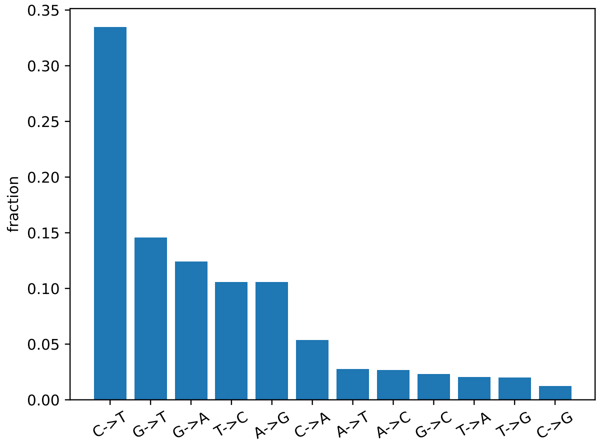

Figure S 2 **The relative rate of different mutations in SARS-CoV-2.** These rates are measured from rare low frequency mutations probably subject to little purifying selection.

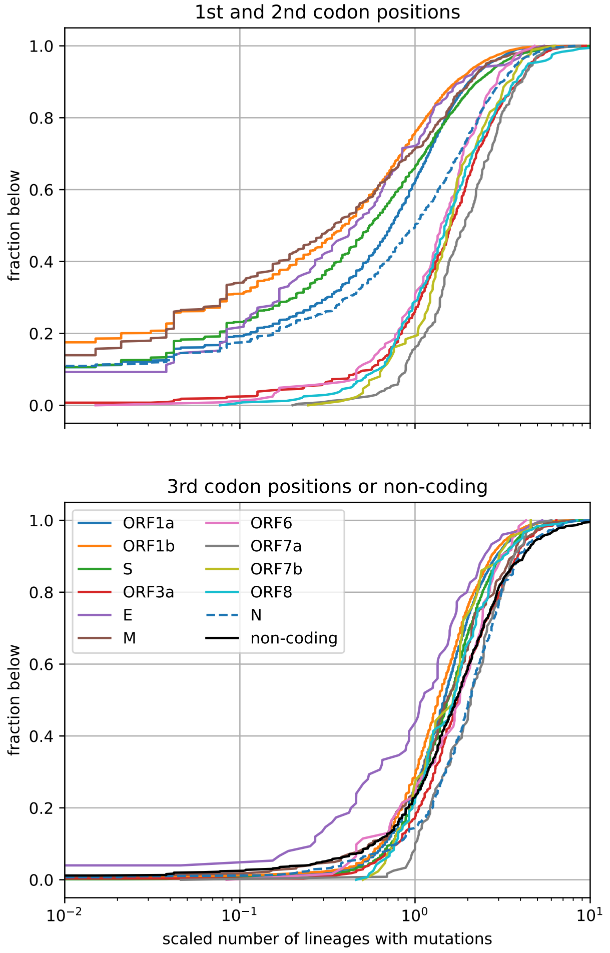

Figure S 3 **Constraints on SARS-CoV-2 mutations by gene.** The top panel quantifies constraint at 1st and 2nd positions in codons of open reading frames. The bottom panel show the analogous distributions at 3rd codon positions. The latter distributions are very similar across genes, with only *E* showing somewhat less variation. In contrast, mutation tolerance at 1st and 2nd positions differs markedly between genes. In ORF3a, ORF6, ORF7a, ORF7b, and ORF8 the distribution of mutations at 1st and 2nd positions is very similar to the distribution at 3rd positions, while the remaining genes show clear signs of constraint.

Appendix: Divergence summaries

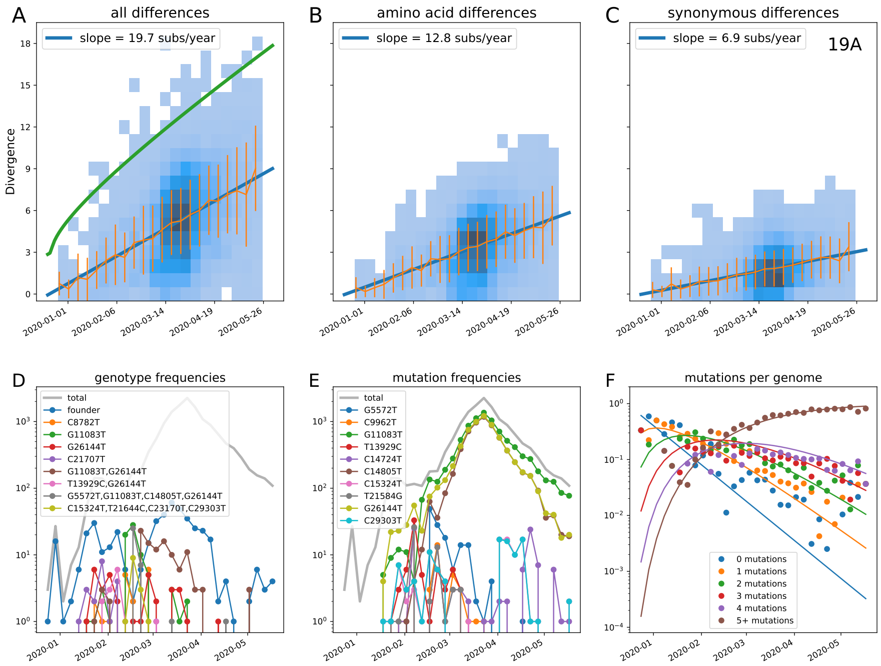

Figure S 4 **Divergence increases linearly with time in clade 19A.** Top: Each panel shows the number of within-clade mutations (total (A), amino acid changing (B), synonymous (C)) as a function of time. The green line in panel A indicates the divergence cut-off, panels B&C only show sequences that pass the divergence filter. Each panel also shows mean ± standard deviation and a weighted linear fit. Analogous figures for all clades considered are included in the appendix. Bottom: Panels D and E show the prevalence of specific genotypes (D) and specific mutations (E). In the case shown, the founder genotype initially dominates and no daughter genotype or mutation dominate. Panel F shows a Poisson model to the breakdown of the population into genotypes with different number of mutations over time.

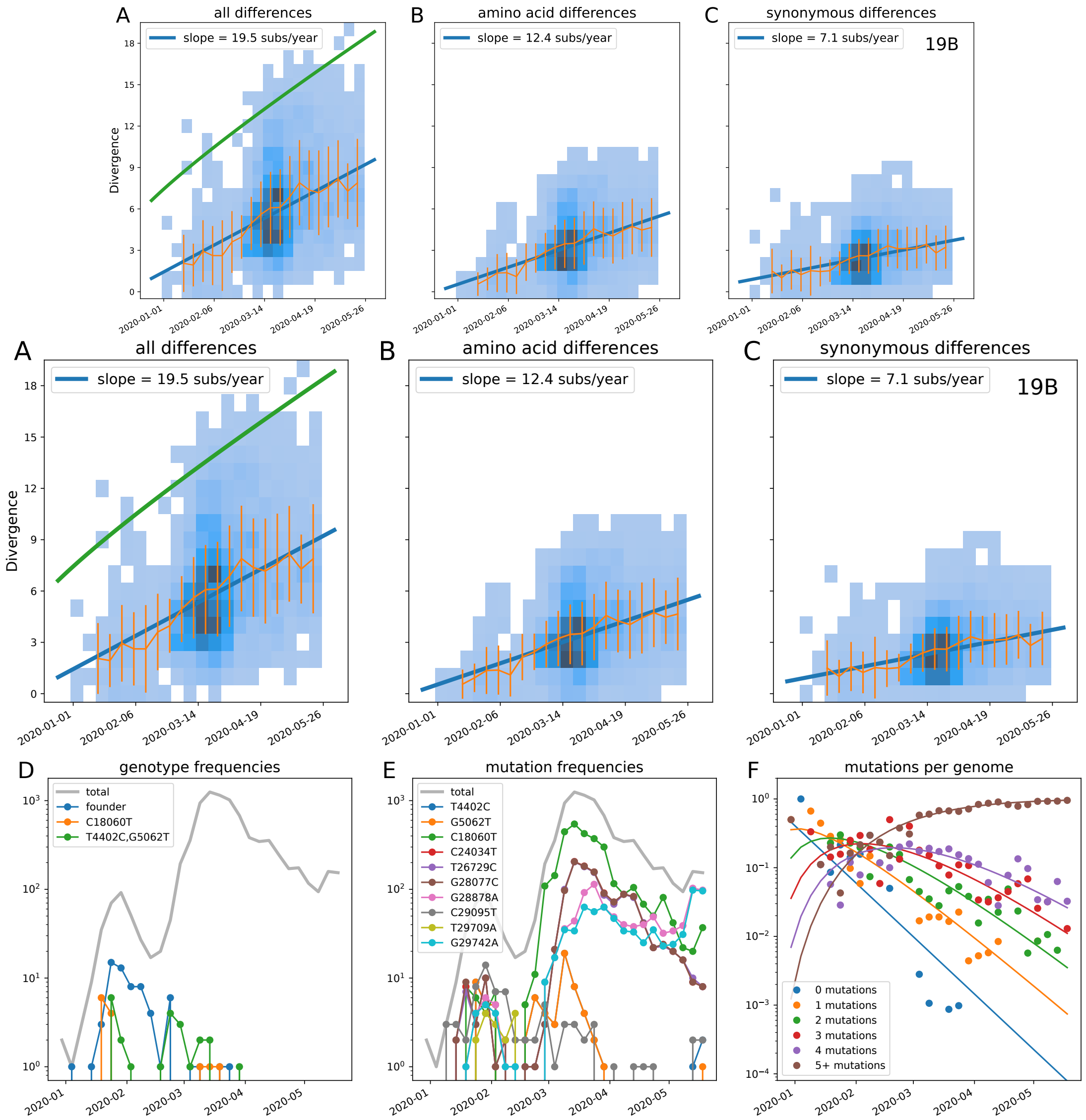

Figure S 5 **Divergence increases linearly with time in clade 19B.**

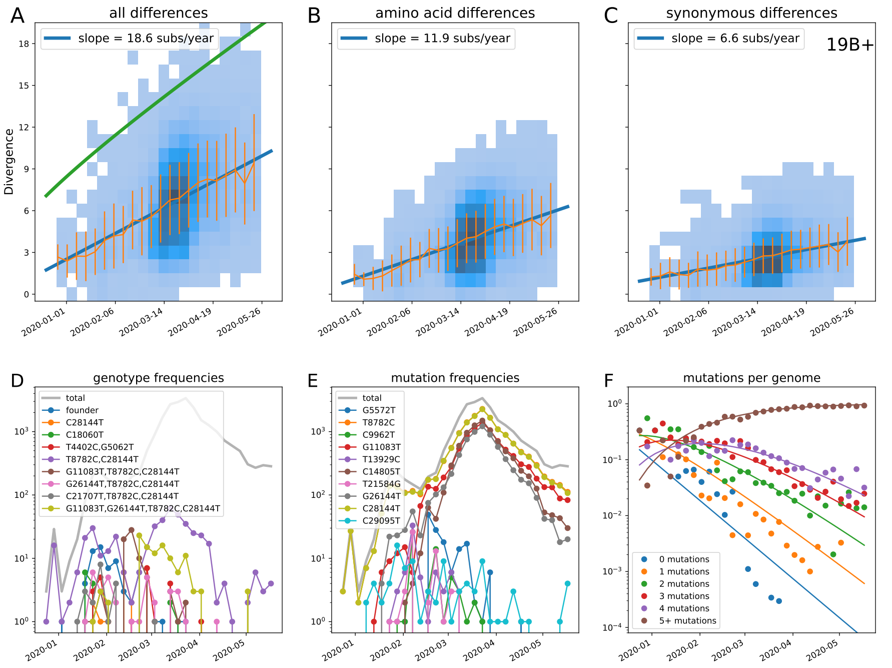

Figure S 6 **Divergence increases linearly with time in clade 19B+.** This figure contains sequences in clades 19 A and B rooted on clade 19B.

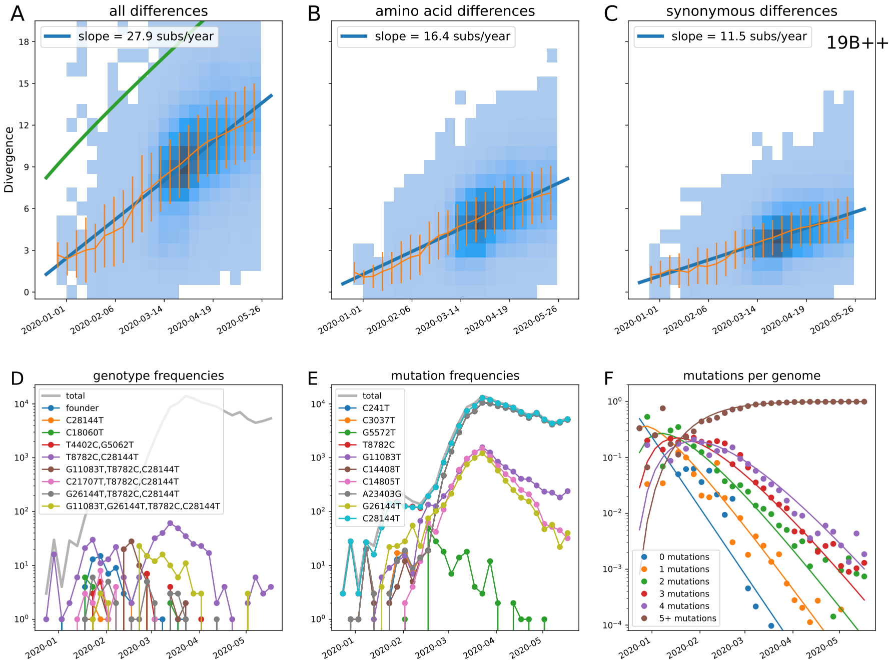

Figure S 7 **Divergence increases linearly with time in clade 19B++.** This figure contains sequences in clades 19A, 19B, 20A, 20B, 20C, and 20D rooted on clade 19B.

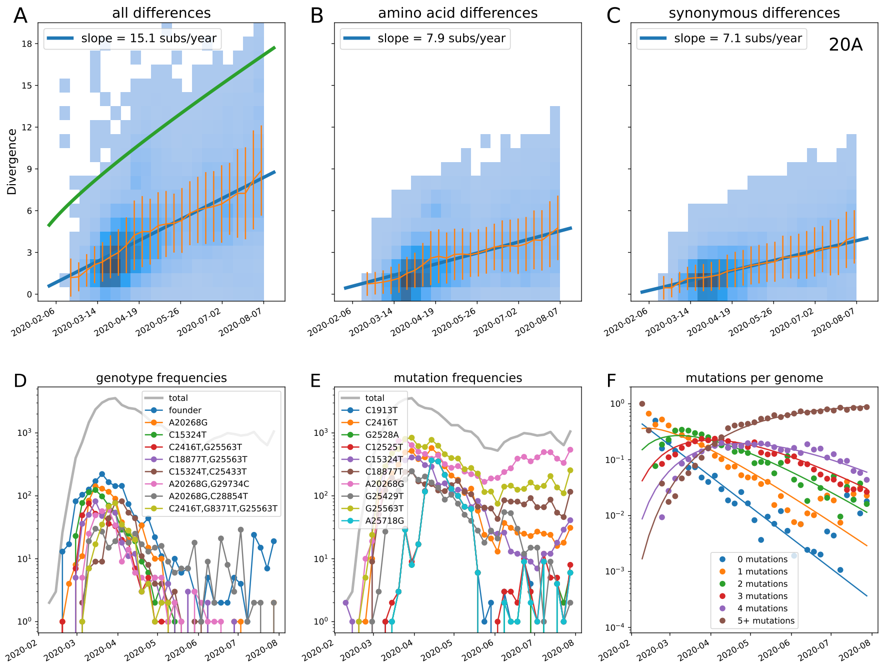

Figure S 8 **Divergence increases linearly with time in clade 20A.**

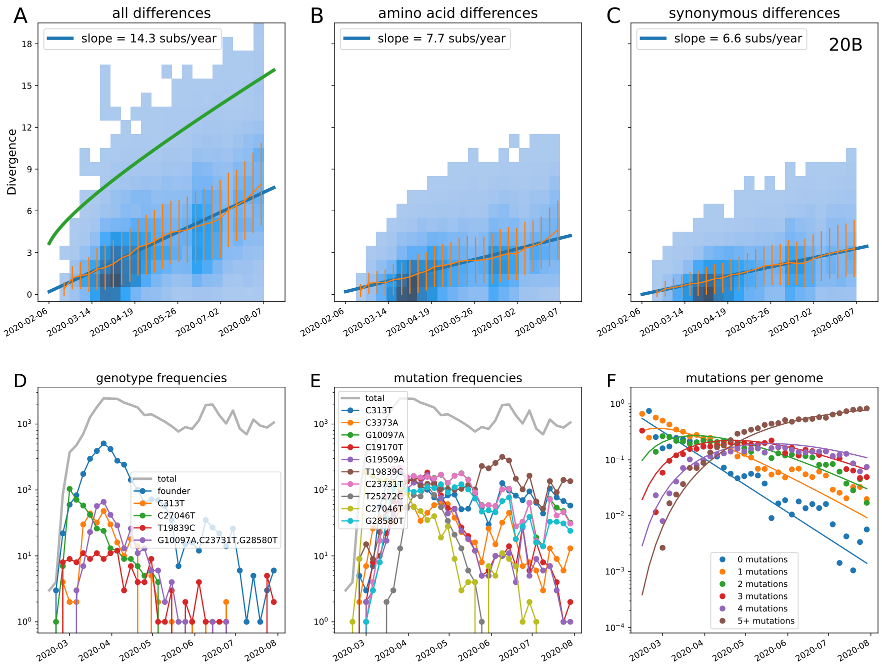

Figure S 9 **Divergence increases linearly with time in clade 20B.**

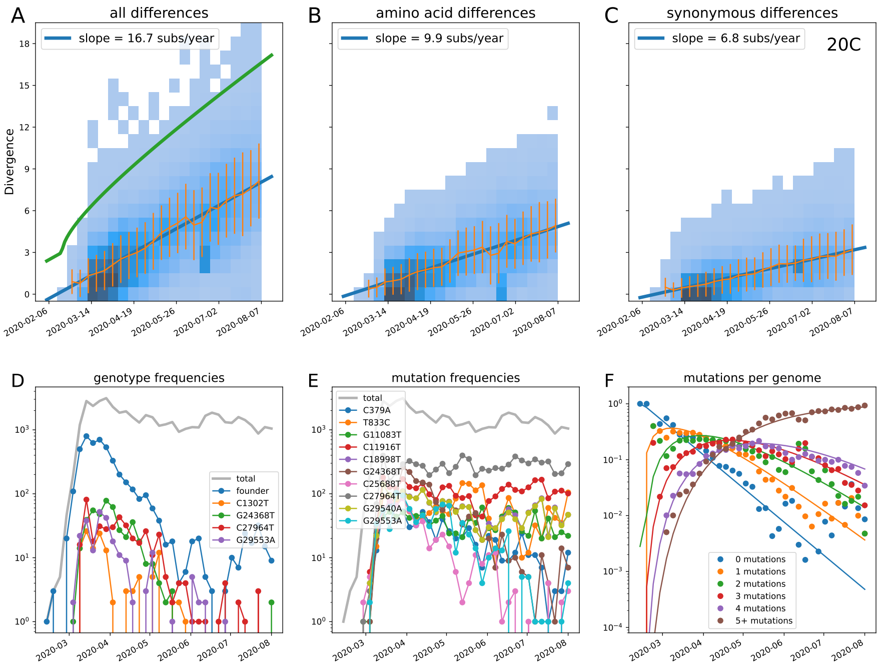

Figure S 10 **Divergence increases linearly with time in clade 20C.**

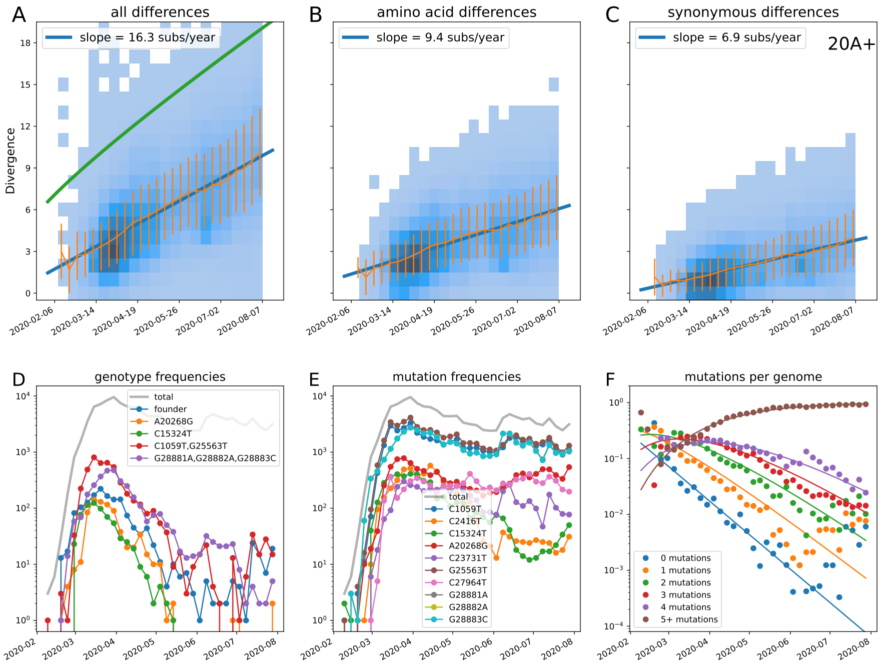

Figure S 11 **Divergence increases linearly with time in clade 20A+.** This figure contains sequences in clades 20A,B,C,D rooted on clade 20A.

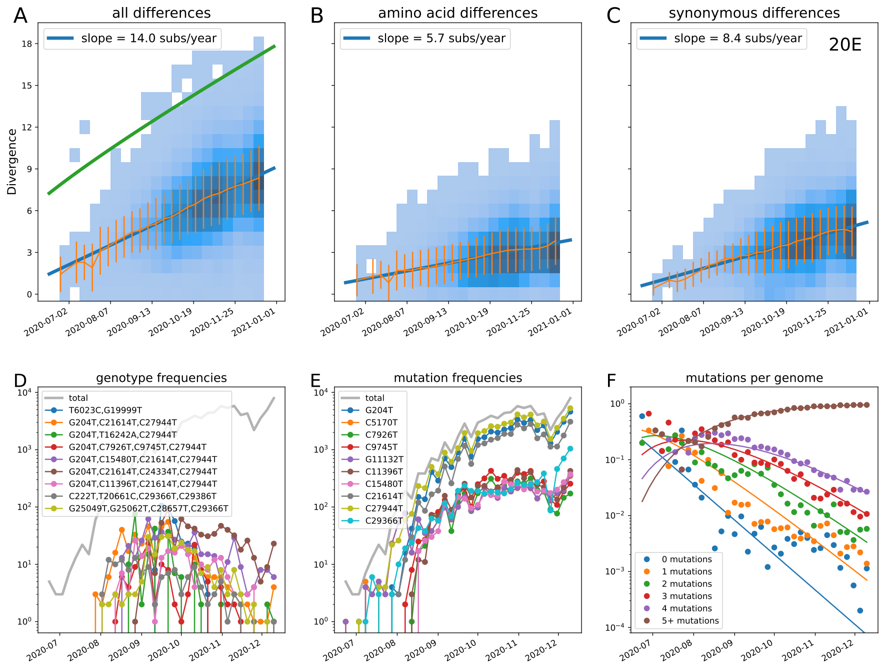

Figure S 12 **Divergence increases linearly with time in clade 20E.**

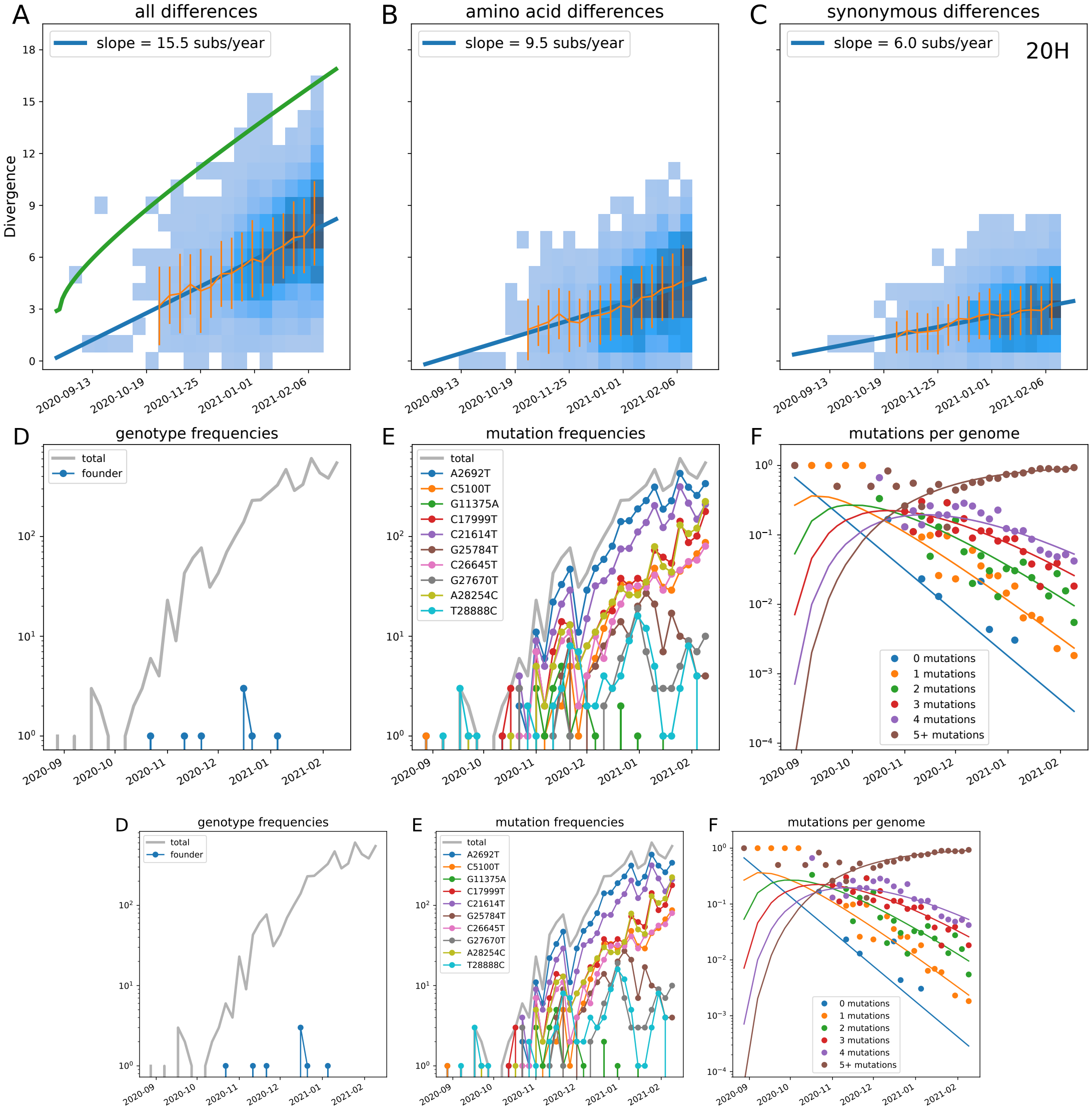

Figure S 13 **Divergence increases linearly with time in clade 20H (Beta).**

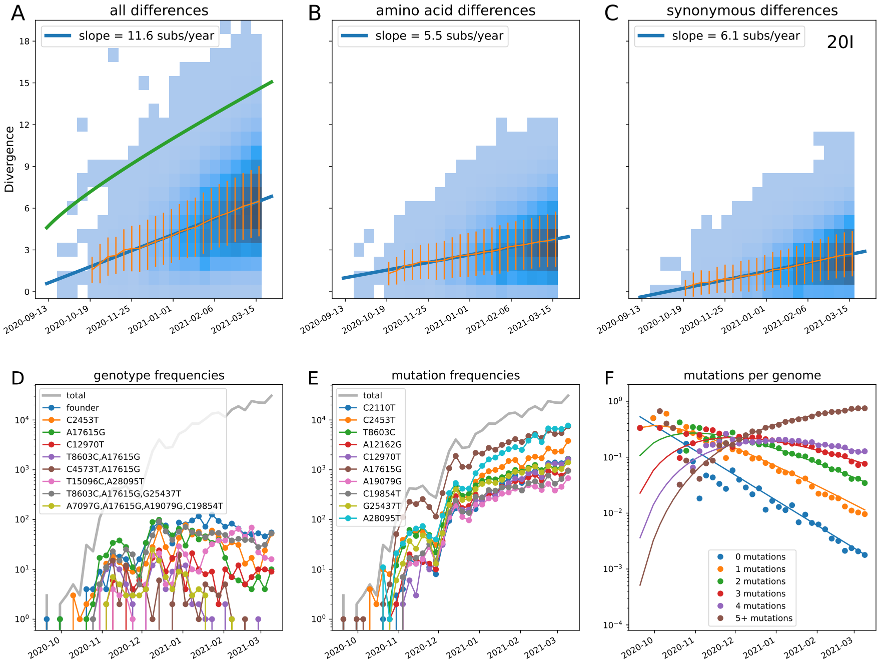

Figure S 14 **Divergence increases linearly with time in clade 20I (Alpha).**

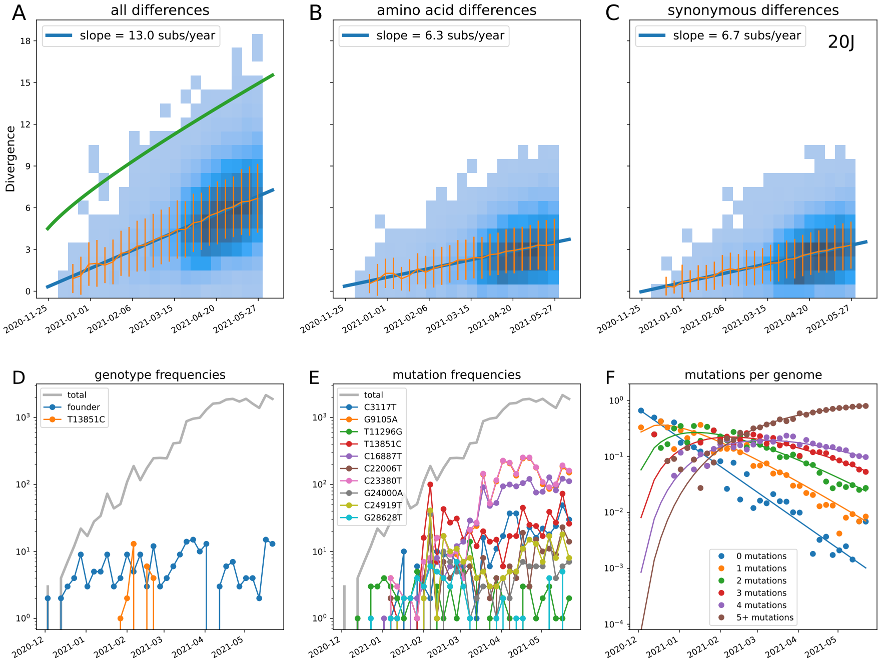

Figure S 15 **Divergence increases linearly with time in clade 20J (Gamma).**

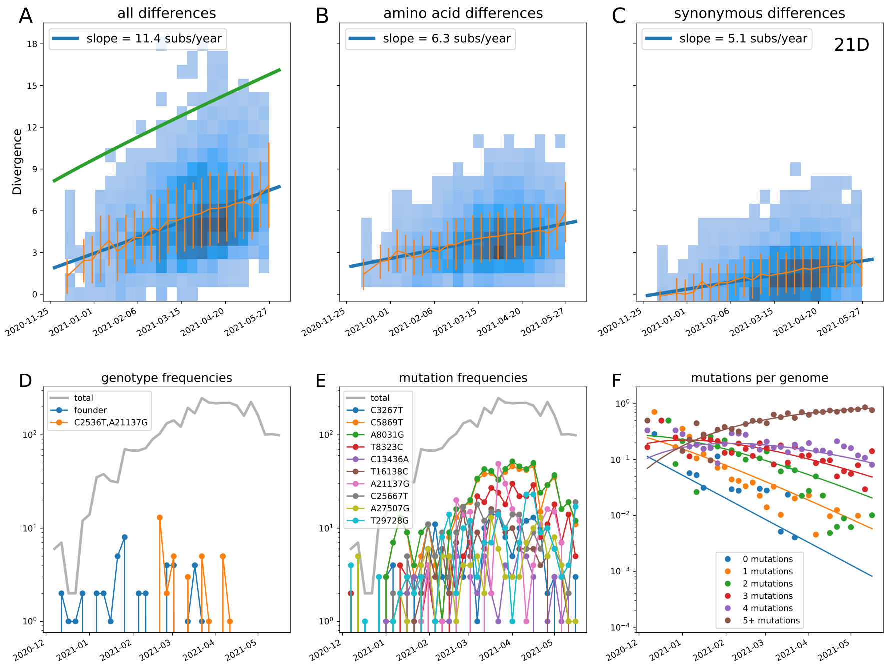

Figure S 16 **Divergence increases linearly with time in clade 21D (Eta).**

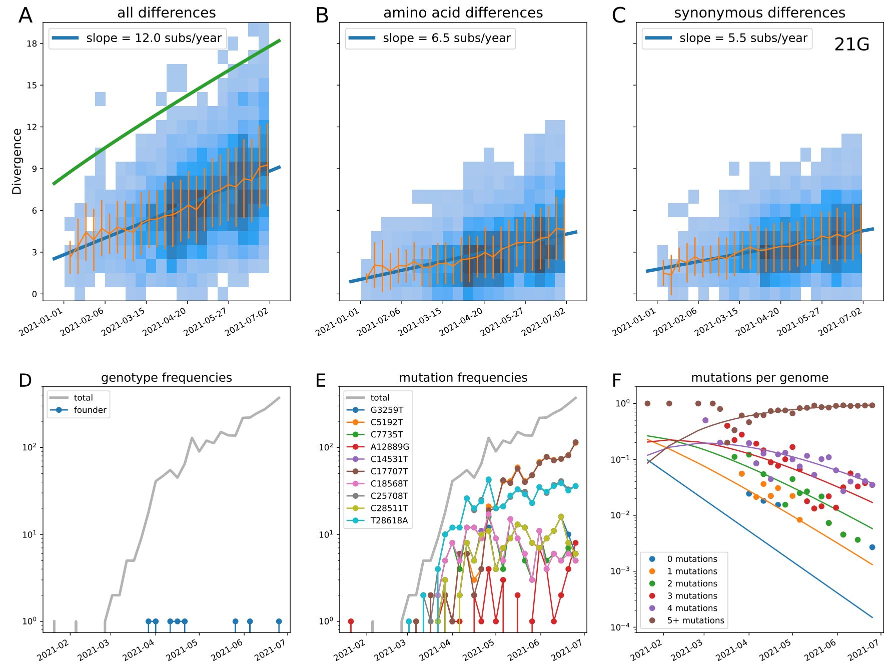

Figure S 17 **Divergence increases linearly with time in clade 21G (Lambda).**

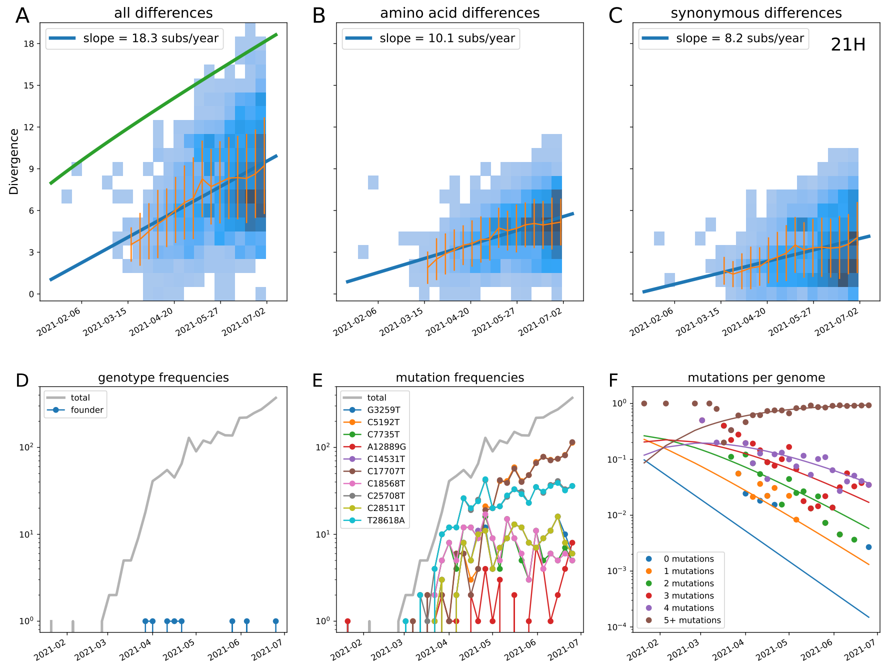

Figure S 18 **Divergence increases linearly with time in clade 21H (Mu).**

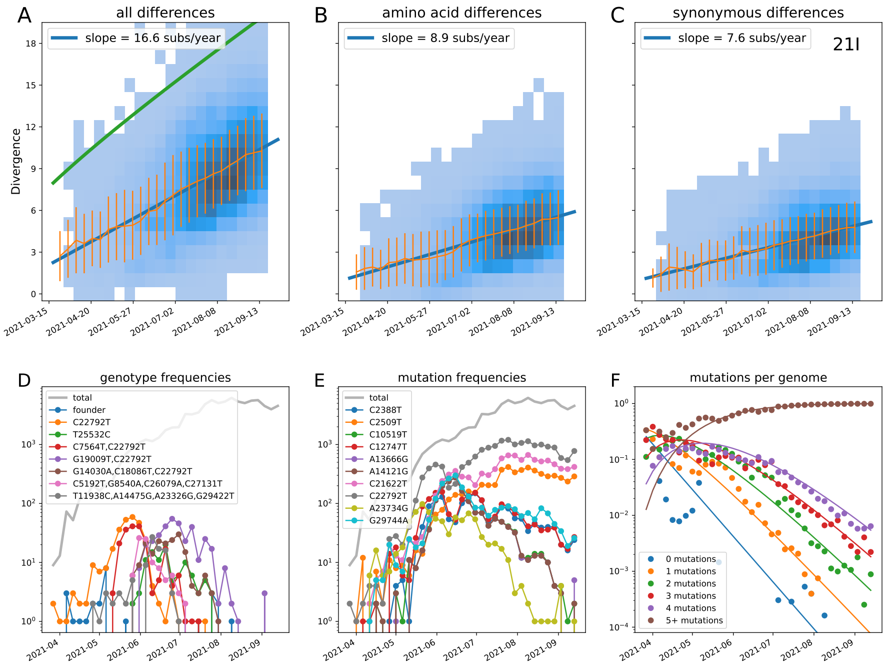

Figure S 19 **Divergence increases linearly with time in clade 21I (Delta).**

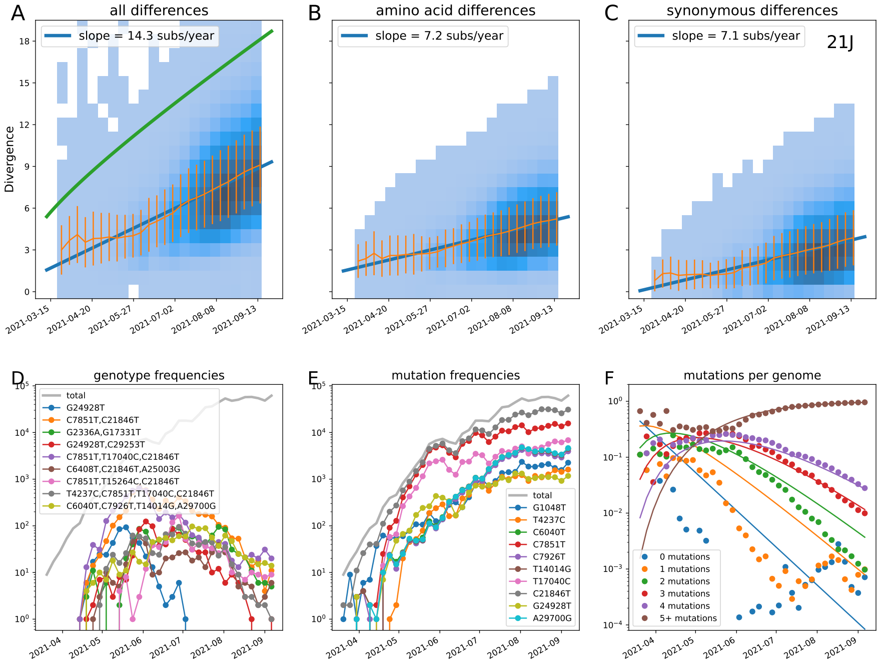

Figure S 20 **Divergence increases linearly with time in clade 21J (Delta).**

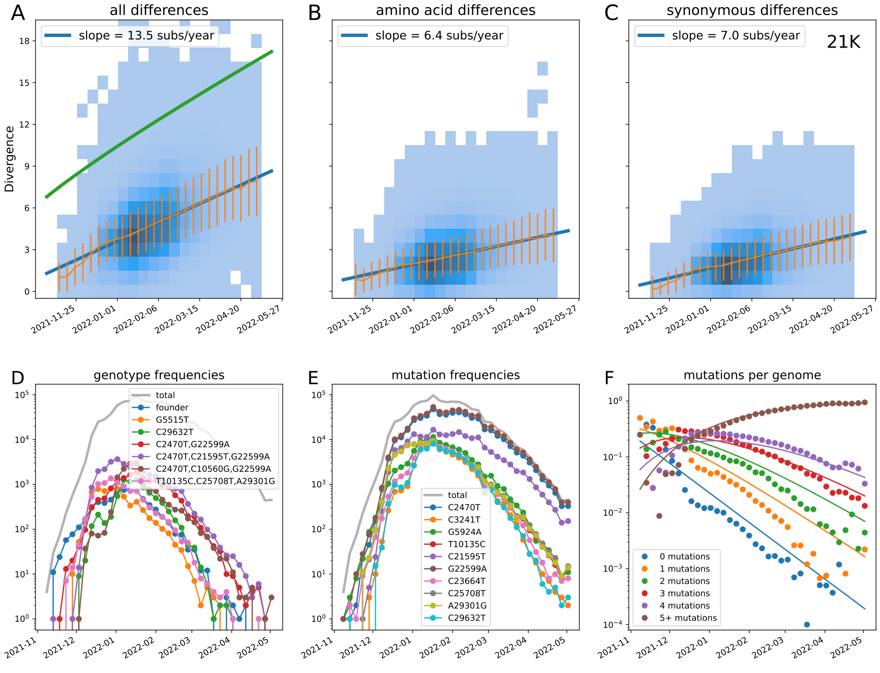

Figure S 21 **Divergence increases linearly with time in clade 21K (Omicron).**

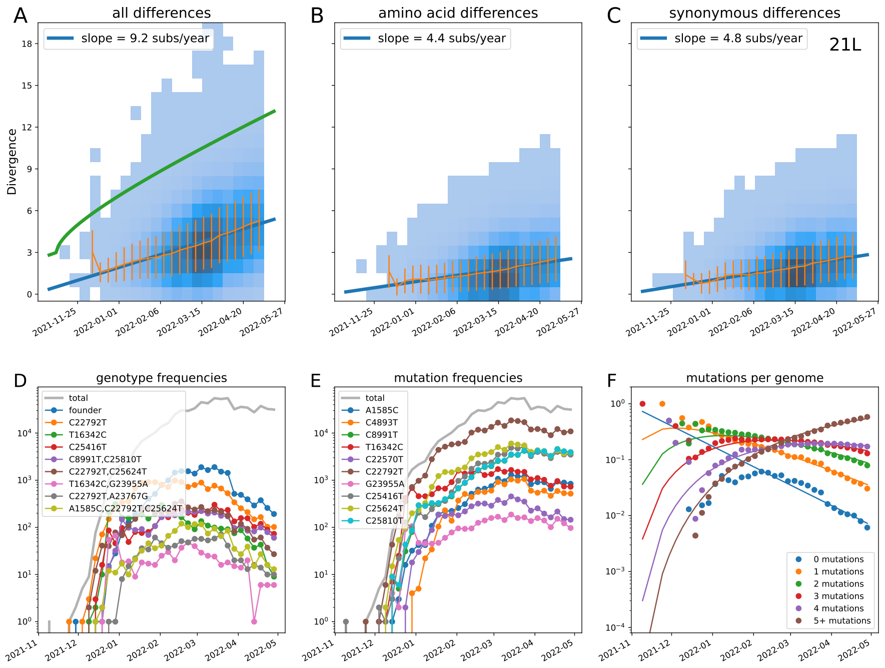

Figure S 22 **Divergence increases linearly with time in clade 21L (Omicron).**

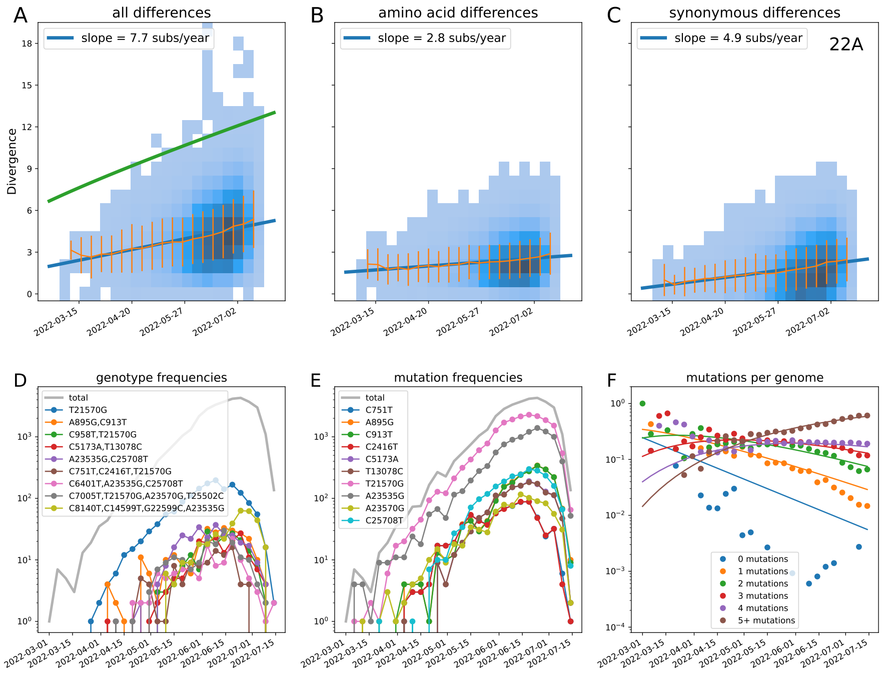

Figure S 23 **Divergence increases linearly with time in clade 22A (Omicron).**

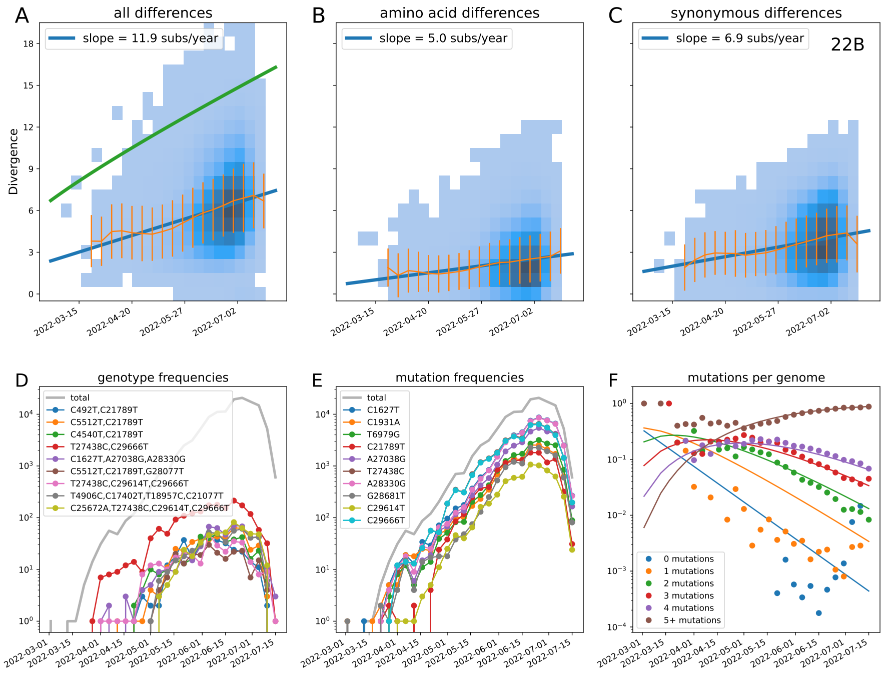

Figure S 24 **Divergence increases linearly with time in clade 22B (Omicron).**
